# Impulsive Action and Impulsive Choice Are Differentially Expressed in Rats Depending on the Age at Exposure to a Gambling Task

**DOI:** 10.3389/fpsyt.2018.00503

**Published:** 2018-10-16

**Authors:** Bo Ram Cho, Myung Ji Kwak, Wha Young Kim, Jeong-Hoon Kim

**Affiliations:** Brain Korea 21 Plus Project for Medical Science, Brain Research Institute, Department of Physiology, Yonsei University College of Medicine, Seoul, South Korea

**Keywords:** impulsive action, impulsive choice, decision-making, rat gambling task, cocaine

## Abstract

Impulsivity is considered an important feature associated with the development of numerous psychiatric disorders, including addictions. In the behavioral approach, impulsivity can be broadly divided into two distinct subtypes: impulsive action and choice. In the present study, we used a rodent version of the gambling task (rGT) to examine how impulsive action and impulsive choice are differentially influenced by difference in age at exposure (i.e., late adolescents/young adults vs. mature adults) to rGT. Rats were trained in a touch-screen chamber to learn the relationships between 4 light signals on the window of the screen and accompanying reward outcomes or punishments associated with different magnitudes and probabilities. Depending on their stabilized pattern of preference when allowed free choice, rats were categorized into risk-averse or risk-seeking group. While undergoing a series of experimental schemes, including extinction, re-acquisition, and acute cocaine injection, rats were re-tested for their premature response during inter-trial interval and choice preference toward the 4 different windows in rGT. Notably, rats exposed early, compared with those exposed late, to rGT showed increased impulsive action, particularly during re-acquisition period, in all sub-groups. In contrast, rats exposed late, compared with those exposed early, to rGT showed increased impulsive choice after acute cocaine injection, but these results were only obtained in a sub-group pre-categorized as high impulsive and risk-averse. These results suggest that different aspects of impulsivity can be differentially expressed during decision-making, and differentially influenced by the age at exposure to a gambling task.

## Introduction

Impulsivity is a common and core feature associated with numerous psychiatric disorders, including attention deficit hyperactivity disorder, substance use disorder, and pathological gambling. It has become increasingly evident that impulsivity is a multi-faceted, rather than a unitary, trait (1–3). In the behavioral approach, the two broadly defined major components of impulsivity are impulsive action and impulsive choice (1–5). Impulsive action is behaviorally manifested as the failure to inhibit an inappropriate response, and consequently, showing premature response. By contrast, impulsive choice is manifested as impulsive decision-making by choosing small immediate rewards over more beneficial delayed rewards. In addition to their behaviorally distinct features, the brain areas mediating impulsive action and impulsive choice are known to be distinct as well (1, 3), and they are also differentially influenced by pharmacological manipulation (4–6), further suggesting that the two components are well-segregated.

In animal studies, impulsive action, expressed as premature response, is widely measured using the 5-Choice Serial Reaction Time Task (5-CSRTT). In contrast, impulsive choice, which involves decision-making, often expressed as devaluing temporally delayed gratification, is frequently measured using delay-discounting task (3, 4, 6). In humans, however, the deficit of decision-making is widely measured using the Iowa Gambling Task (IGT), which simulates real-life decision-making by adding the features of reward, punishment, and uncertainty (3). Similarly, adopting the basic principle of IGT, the rodent version of the gambling task (rGT), which shares many of the features of the human gambling tasks (7), has been developed by a few research groups (8–10). Recently, we adopted one of the previously developed rGT models (10), with a modification of the touch-screen chamber (11), and successfully trained rats to demonstrate decision-making toward risk-preference (12). In rGT, for rats to be trained to perform decision-making behaviors with gambling features, they require pre-training steps with multiple stages, one of which is very similar to the 5-CSRTT. Thus, rGT provides experimenters the advantage of measuring impulsive action and impulsive choice simultaneously in a within-subjects frame.

Adolescence is an extremely important period in development, during which the brain matures and higher order cognitive functions develop to shape adjustable normal behaviors. This period is also vulnerable to the development of many neuropsychiatric disorders and remarkably more prone to risk-taking behavior and impulsiveness (13–15). These behavioral characteristics of adolescence further interact with environmental factors (e.g., stress and drugs of abuse) to determine the onset of neuropsychiatric disorders (13). When considering the laboratory rat, however, it is difficult to precisely compare rat and human age across the different stages of life. Albeit based on a limited number of studies, it is generally considered that approximately postnatal day (PND) 28, after weaning, is the beginning of adolescence, and PND 63–70 is the period when male rats enter into adulthood (13, 16).

Although the two major components of impulsivity are known to exist in segregation, there have been relatively few studies examining their relationship with each other and their differential expression when rats are placed under stressful situations, such as extinction and re-acquisition of a pre-established task; in particular, there is a lack of studies comparing these parameters across different developmental transition periods. To address these issues, in the present study, we exposed rats to rGT at two different ages (i.e., late adolescent/young adult vs. mature adult), and assessed how impulsive action and impulsive choice are expressed under different situations. In addition, as there is ample evidence that maladaptive decision-making is associated with an increase in cocaine usage in both humans and animals (17–20), we also examined how acute cocaine administration influences the expression of impulsivity.

## Materials and methods

### Animals

Male Sprague-Dawley rats [Crl:CD(SD); PND 21] were obtained from Orient Bio Inc., (Seongnam-si, Korea). The rats were housed three per cage (GR900; 21.3 cm high × 34.6 cm long × 39.6 cm wide; Tecniplast Inc., Buguggiate, VA, Italy) for 1 week to allow habituation to a new colony environment, during which they were handled by experimenters, and had access to food *ad libitum*. Subsequently, they were housed two per cage and simultaneously placed on a restricted diet with 85% of their normal daily food consumption, which was started 2 days before the pre-training experiments and maintained until the end of experimentation. Food was provided immediately after the daily training session to sufficiently maintain the animals' growth and motivation. Water was available *ad libitum* at all times. Colony rooms had a controlled room temperature (21°C) and a 12 h light/dark cycle (lights on at 8:00 am), and all experiments were conducted during the day. All animal use procedures were conducted according to an approved Institutional Animal Care and Use Committee protocol of Yonsei University College of Medicine.

### Drugs

Cocaine hydrochloride was purchased from Belgopia (Louvain-La-Neuve, Belgium). It was dissolved in sterile 0.9% saline to a final concentration of 15 mg/ml.

### Apparatus

**Figure 1:**
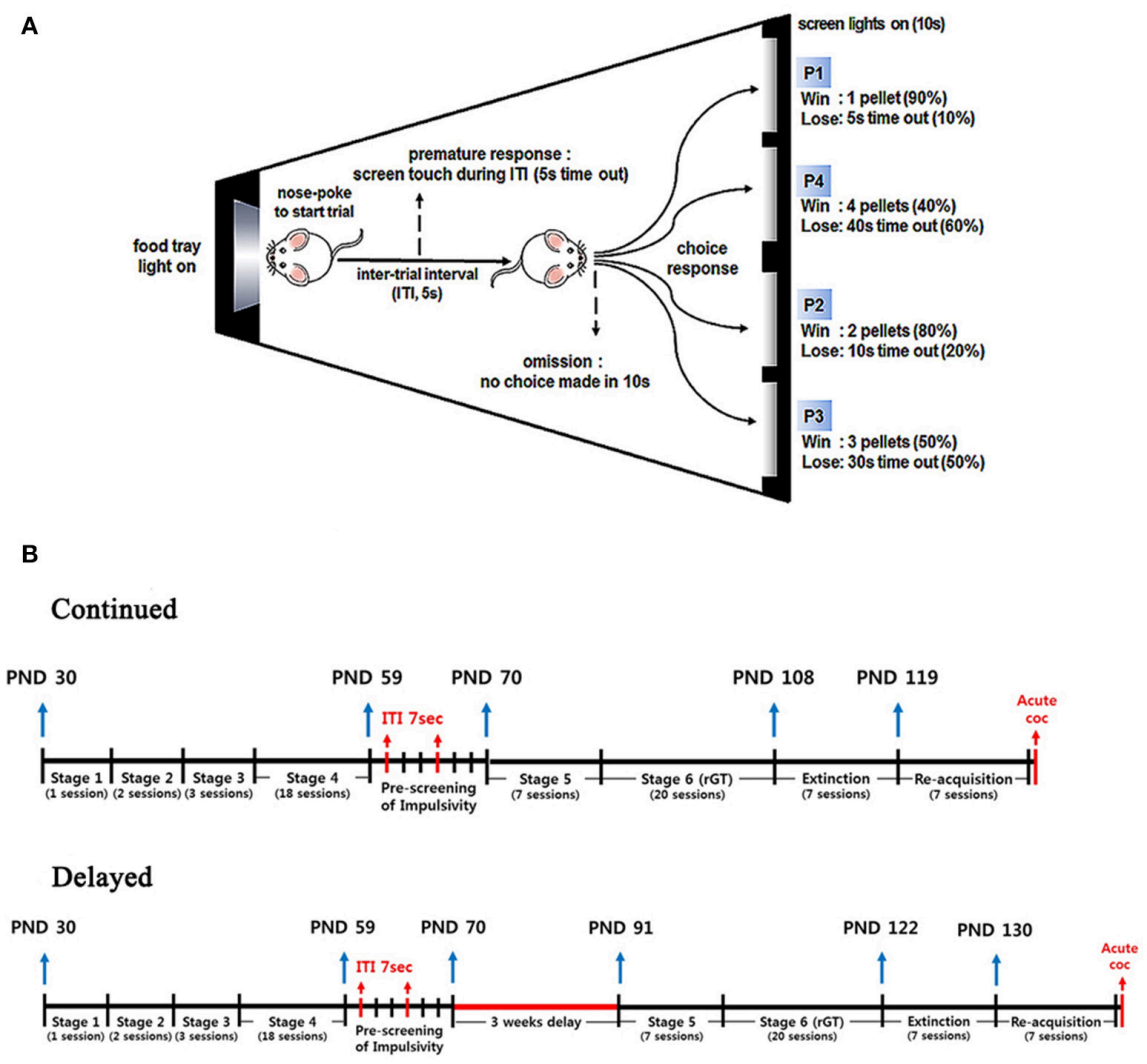
Schematic diagram for the rGT chamber and time lines for the whole experimental procedures. **(A)** Schematic diagram of the rGT chamber, where a food magazine unit (left) and 4 response windows (right) are shown. Each window is represented as P1 through P4 with a different number of pellets, duration of time-out, and frequencies. **(B)** Time lines for the whole experimental procedures were illustrated with rat's age indicated. A single session can be added to the age as 1 day. Note that there were days with no experiments conducted during the weekends.

The rGT was conducted in a set of eight identical touchscreen-based automated operant chambers housed in dense sound- and light-attenuating boxes (68.6 cm high × 60.7 cm long × 53.5 cm wide; Campden Instruments Ltd., Leics, UK). Each chamber was equipped with a house light (light-emitting diode), touch-sensitive liquid crystal display monitor (touchscreen; 15.0 inch, screen resolution 1,024 × 768), pellet dispenser, and food magazine unit (with light and infrared beam to detect entries) facing the touchscreen. The chambers had a trapezoidal shape [30 cm high × 33 cm long (from screen to magazine)×25 cm wide for the screen and 13 cm wide for the magazine; Figure 1A], which was designed to help focus the animal's attention on the touchscreen and reward delivery area (i.e., the food magazine) (11). On top of the chamber, a transparent lid was secured to the trapezoidal walls with latches to retain the animals inside the chambers. The floor was constructed from perforated stainless steel, and a tray for collecting litter was located below the floor. The touchscreen used sensitive optical infrared sensors that allowed the screen to reliably detect an animal's touch without pressure. A black plastic mask (36 cm high × 28 cm wide) with five response windows (the size of each window was 3.0 cm high × 3.0 cm wide, positioned in a row with the windows spaced 1.0 cm apart, 3.5 cm from the grid floor) was fitted in front of the touchscreen, which helped reduce accidental screen touches and clearly distinguish the response locations from the background. The visual stimulus, a solid white square, was shown only through the two left and two right response windows, the middle window was left black. We used the Whisker Standard Software (Campden Instruments, Ltd., Leics, UK) (21) as the controlling software, and the four chambers were controlled using two computers each.

### rGT pre-training

Pre-training methods have been described in detail in our previous study (12). In brief, animals were trained once daily in a 30 min session, 5 days per week. Sucrose pellets (45 mg) with chocolate flavor (Bio-Serve, Flemington, NJ, USA) were used as a reward. In stage 1, the animals were first habituated to the touchscreen chamber for one session. In stages 2 and 3, which lasted over five daily sessions, animals were trained to learn the relationship between the light stimulus on the screen and the reward pellet, and to touch the screen to receive a pellet as a reward. In this stage, the inter-trial interval (ITI) of the 5 sec rule was first applied such that animals had to wait for 5 sec after pushing their noses into the food magazine to start a new trial. In stage 4, which lasted over 16 to 18 daily sessions, animals serially learned to touch one of the four windows which were randomly lit, within different stimulus durations (starting from 60 sec, then serially reduced to 30, 20, and finally, 10 sec), to receive one pellet. Animals completed the task either within 100 trials or 30 min, whichever came first. In this stage, they learned for the first time that they were punished with a time-out (i.e., the white house-light was lit for 5 sec) if they touched the screen without waiting during ITI (premature) or if they did not touch the screen within the stimulus duration (omission). They were also punished if they touched other windows which were not lit. When the accuracy was >80% and omissions were fewer than 20%, the animals were considered to have acquired the task (i.e., with an ITI of 5 sec and a stimulus duration of 10 sec).

### rGT training

Essentially, during rGT training, the animals were confronted with four choices differing in their probability and magnitude of reward (food) and punishment (time-out), and they had to learn an optimal strategy to determine the choice that provided the most reward per session (10). In stage 5, which lasted over 7 daily sessions, the animals learned for the first time the relationship between each window and the reward/punishment ratio assigned to that window, which was as follows: window (P1), 1 pellet (90%) or 5 sec time-out (10%); window (P2), 2 pellets (80%) or 10 sec time-out (20%); window (P3), 3 pellets (50%) or 30 sec time-out (50%); and window (P4), 4 pellets (40%) or 40 sec time-out (60%). In this stage, one of the four windows was randomly lit for 10-sec and animals were punished (i.e., the white house light was lit for 5 sec) for a premature response. Additionally, for the first time in this stage, animals were punished (time-out; i.e., the white house light was lit, and all the windows on the screen simultaneously flashed for 5 to 40 sec) even on correctly touching the screen according to the pre-designated schedule for each window. So far, from stages 1 to 5, only one of the four windows on the screen was randomly lit. However, in stage 6, all four windows were simultaneously lit when each new trial started, and animals were allowed to wait for an ITI of 5 sec of and then choose one of the four windows, which were lit for 10 sec. The reward and punishment settings designated for each window were the same as those introduced in stage 5. Depending on which window the animals chose, they would receive either reward (pellet) or punishment (time-out) with differently programmed probabilities. Once a trial was finished, regardless of the outcome, they again encountered four different choices in the next trial, and this process was repeated for 30 min. Hypothetically, if one window was chosen exclusively, the amount of reward pellets per session that an animal could obtain was as follows: P1, 295; P2, 411; P3, 135; and P4, 99 pellets (22). The percentage of choices [(number of choices for a specific window divided by the total number of choices made) × 100] was used to measure the animals' preferences for the different windows. After 20 daily sessions were completed, the average of the last three daily sessions' choice percentages was considered a basal score for the animals' risk-preference. Animals were categorized as risk-averse when their basal score for P2 (the most optimal choice) was equal to or higher than 60%, whereas they were categorized as risk-seeking when it was lower than 60%. To avoid any location bias, windows were allocated in a counterbalanced way as follows: for half of the animals, the windows were 1 (P1), 2 (P4), 3 (P2), and 4 (P3); for the other half of the animals, the windows were 1 (P4), 2 (P1), 3 (P3), and 4 (P2). In addition to premature response and omission (both were expressed as a percentage of the total number of trials initiated), choice-related behavioral parameters, such as choice response [(number of times the window was correctly touched divided by the total number of trials initiated) × 100], perseverative response (repeatedly touching the screen during punishment, calculated as the total number of screen touches divided by the total duration of punishment), feed-tray entry [repeatedly entering the food magazine, calculated as the number of feed-tray entries divided by the number of trials (including omissions) × 100], reward collection latency (the time required for animals to obtain the reward after a correct screen touch), and correct response latency (the time required for animals to correctly touch the screen, after the end of the ITI, while the screen was lit) were analyzed.

### Pre-screening of impulsivity

Following successful acquisition of the stage 4 task, with an ITI of 5 sec, rats were further tested to examine their trait impulsivity (impulsive action) in a modification of stage 4, consisting of two daily sessions with an ITI of 5-sec, followed by a session with an ITI of 7 sec, repeated twice consecutively to amplify the appearance of premature response (23). With the mean score of premature responses obtained from the measurements for the two sessions with ITI of 7 sec, rats were categorized as high impulsive (HI) if they scored above the standard error of the total group mean, and as low impulsive (LI) if they scored below. Rats which scored within the standard error were excluded from the subsequent experiments.

### Design and procedures

A schematic illustration showing time lines for the whole experimental design was depicted in Figure 1B.

Two days after starting the food restriction, all rats (*n* = 64) were serially trained in stages 1 to 4. Once they were categorized as HI (*n* = 30) and LI (*n* = 28), they were sub-divided into continued or delayed groups, resulting in four groups, i.e., HI-continued (*n* = 15), LI-continued (*n* = 13), HI-delayed (*n* = 15), and LI-delayed (*n* = 15). Rats in the continued group were continued undergoing training into stages 5 and 6 without delay after stage 4, while those in the delayed group were not trained for 3 weeks, after which their training into stages 5 and 6 was resumed. Thus, rats were exposed to rGT at different times. By the time they reached stage 5, rats in the continued group were ~10 weeks old, while those in the delayed group were 13 weeks old.

Once the rats had completed all the sessions in stage 6, they were further sub-divided into risk-averse or risk-seeking groups according to the average score for P2 for the last 3 days of stage 6 training, resulting in eight different sub-groups, i.e., HI-continued-averse (*n* = 7), HI-continued-seeking (*n* = 7), LI-continued-averse (*n* = 5), LI-continued-seeking (*n* = 7), HI-delayed-averse (*n* = 7), HI-delayed-seeking (*n* = 7), LI-delayed-averse (*n* = 5), and LI-delayed-seeking (*n* = 8). A total of 5 rats (1 each from HI-continued, LI-continued, and HI-delayed, and 2 from LI-delayed) which had undergone fewer than five trials were excluded from all further analyses, and finally, results from 53 rats were used for analyzing the data presented herein.

After completion of stage 6, rats underwent extinction, comprising a total of seven daily sessions, in which they performed the same task as stage 6, but did not receive reward pellets. All other parameters were unchanged. Choice responses and omission (%) were used to assess whether extinction had occurred. After completion of extinction, the rats underwent re-acquisition comprising of seven daily sessions, in which they were re-exposed to the stage 6 task, with the reward pellets made available again. Finally, after completion of re-acquisition, the rats performed a single session of the stage 6 task following acute intraperitoneal administration of cocaine (15 mg/kg) 30 min before performing.

### Statistical analysis

Data are shown as mean + standard error of the mean, and they were analyzed using Sigma Plot, version 12.5 (Systat Software, Inc., Chicago, IL, USA). An arcsine transformation was performed for the data obtained as percentages before the analysis. The data were analyzed using two-way analysis of variance (ANOVA), with or without repeated measures, followed by *post-hoc* Bonferroni comparisons. ANOVA was validated by both normality and equal variance tests. Differences between experimental conditions were considered statistically significant when *p* < 0.05.

## Results

### Pre-screening of impulsivity

**Figure 2:**
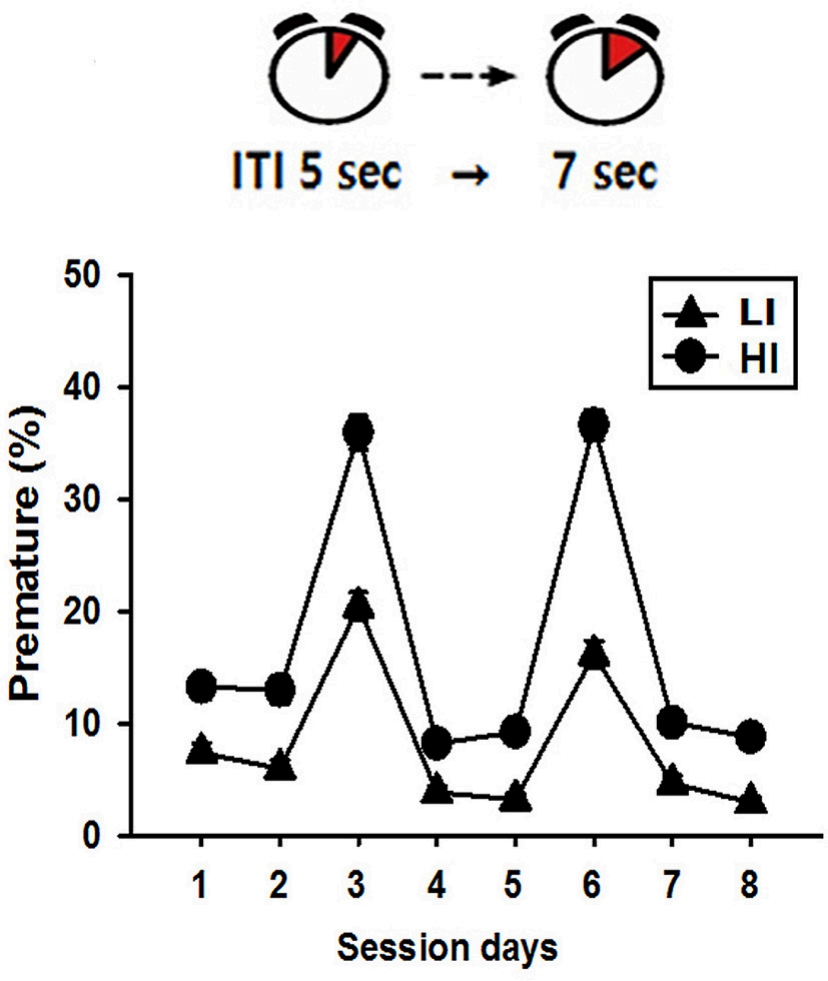
Pre-screening of impulsivity scores. The mean values of premature response scores during daily sessions indicate that there were clear difference between LI and HI, especially when ITI was changed from 5 to 7 sec on session day 3 and 6.

When allowed to wait for 7 sec, rather than the normal 5 sec, before touching the lit window on the screen, rats could be clearly categorized into LI and HI groups, according to their mean premature response scores of the duplicate measurements. Out of the total 64 rats, the mean ± standard error of the premature response score as a percentage was 27.53 ± 1.19%. Rats were categorized as HI if their mean scores were higher than the boundary of standard error of the total group mean (i.e., 28.72%), and as LI if their mean scores were lower than the boundary of standard error (i.e., 26.34%). After categorization, the mean values for the LI and HI groups were 18.25 and 36.25, respectively. Figure 2 shows the mean values of the premature response scores during daily sessions.

### Effect of difference in age at exposure to rGT on basal scores for risk-preference

**Figure 3:**
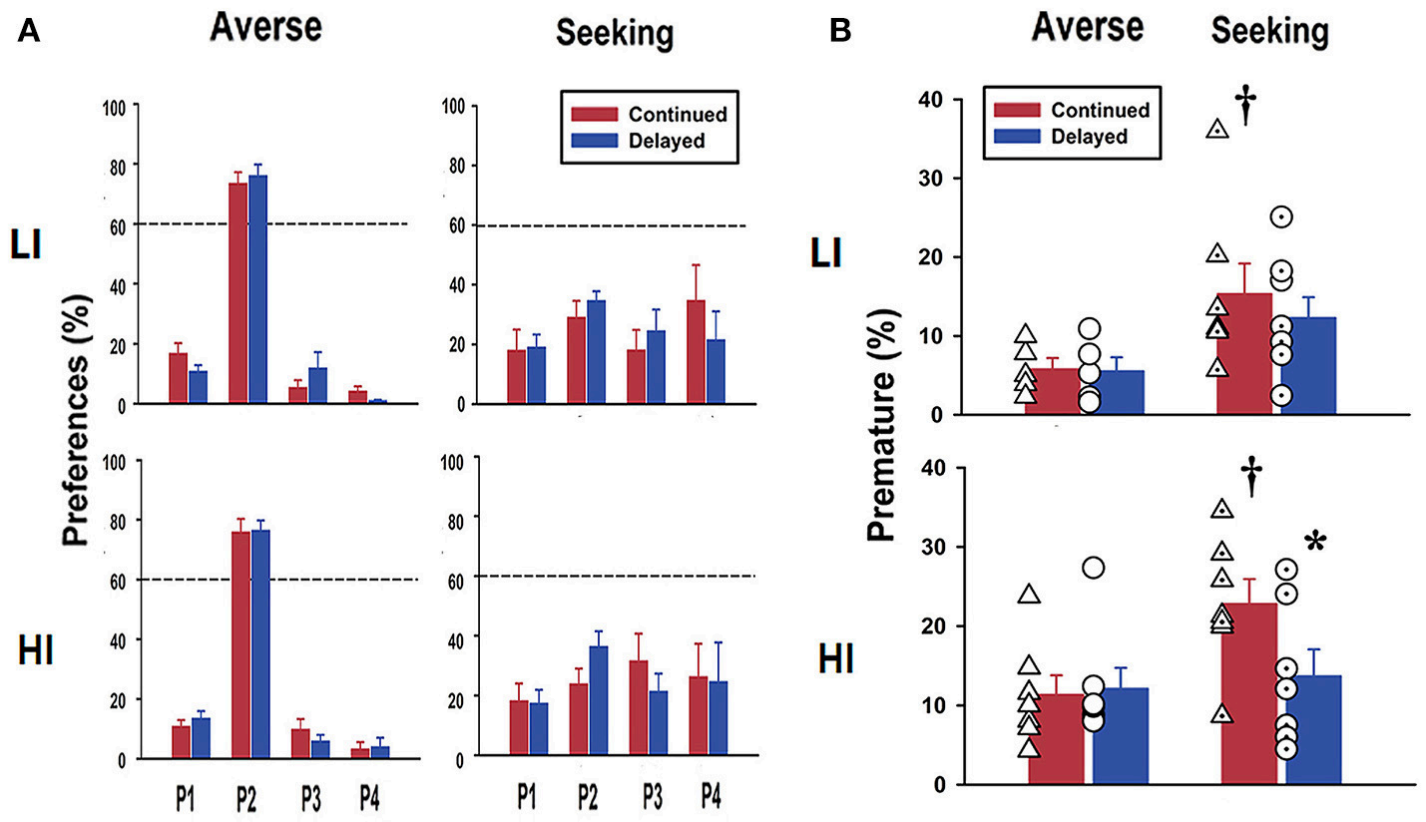
Basal scores for risk preference and premature response scores after rGT. **(A)** Data obtained after rGT training clearly show different risk preferences between the groups. The risk-averse group overwhelmingly chose P2 over the other windows more than 60% of the choices, whereas the risk-seeking group chose P2 <60% of the choices. There were no significant differences between LI and HI groups. The continued group also made no differences when compared to the delayed group. **(B)** Premature response scores obtained after rGT show that the HI group had higher scores than the LI group, and it was the same for the risk-seeking group compared to the risk-averse group. Within the same risk-seeking group, the continued group had significantly higher increased scores for premature response than those in the delayed group, only in pre-categorized as the HI group. Values are expressed as a mean+standard error of mean. *p < 0.05; compared to continued group within risk-seeking. ^*†*^*p* < 0.05; compared to risk-averse within the continued group. Individual scores are shown as symbols (triangles and circles for continued and delayed group, respectively) overlaid on top of the bar graph.

After completion of stage 6, rats were separated into risk-averse and risk-seeking groups, depending on their stabilized preference for P2 being above or below 60%, respectively (Figure 3A). There were no differences in the preferences toward risk choice between the LI and HI groups. Further, the continued and delayed groups did not exhibit differences in preferences. When we analyzed premature responses, it was found, as expected, that the HI group had higher scores than the LI group, regardless of their preferences. This was also the case when the risk-seeking group was compared with the risk-averse group (Figure 3B). The results of the two-way ANOVA conducted on these data showed a significant effect of risk preference [risk-averse vs. risk-seeking; *F*_(1, 24)_ = 7.76, *p* < 0.011]. Interestingly, within the risk-seeking group in the pre-selected HI group, the continued group showed significantly higher scores than the delayed group for premature response revealed by *post hoc* Bonferroni comparisons (*p* = 0.033; bottom panel in Figure 3B).

### Re-acquisition after extinction and acute cocaine administration differentially modifies premature responses and risk preference depending on the age at exposure to rGT

**Figure 4:**
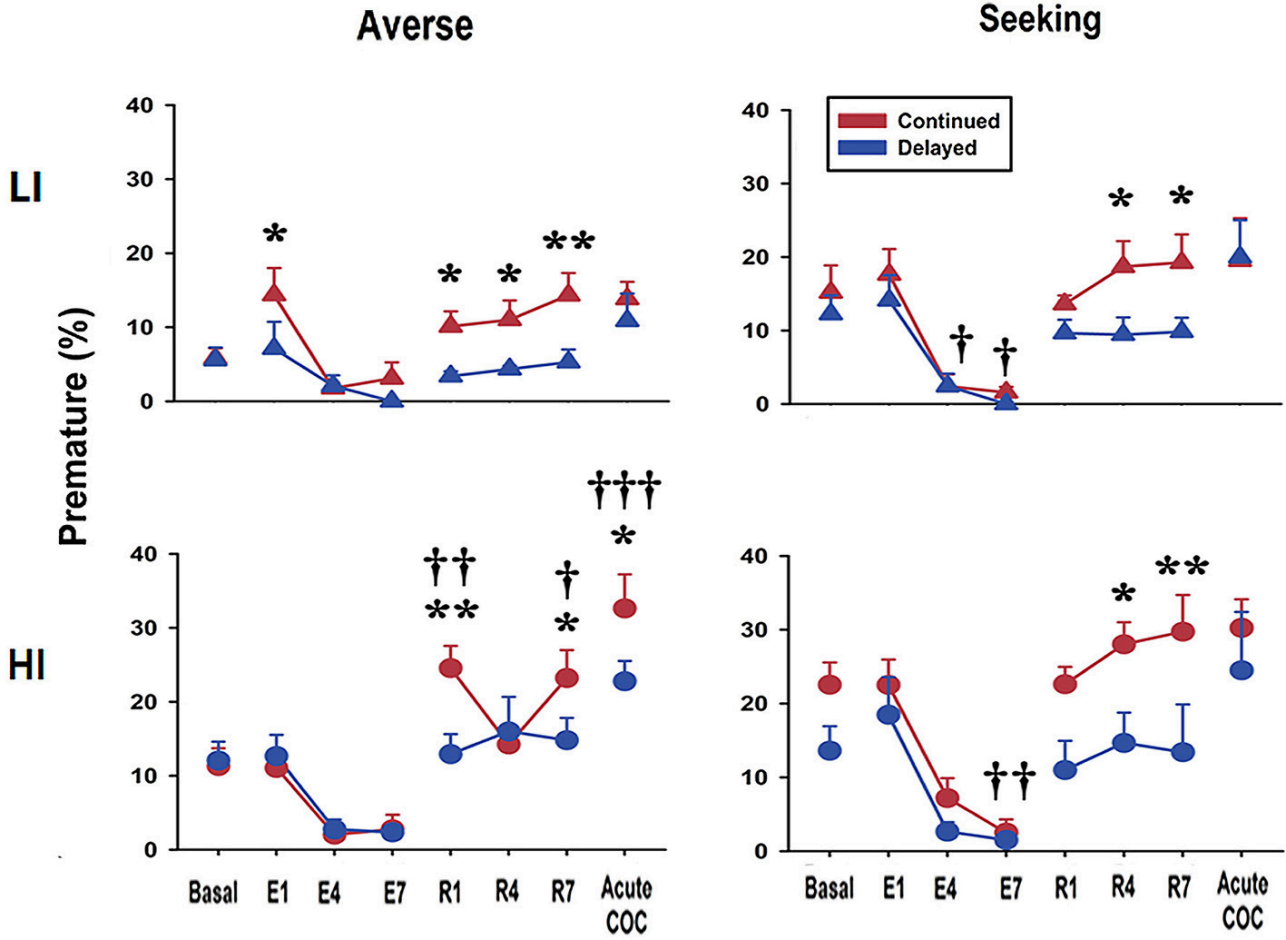
Premature response scores during extinction, re-acquisition, and after acute cocaine injection. Premature response scores obtained during re-acquisition period show that the continued group had higher scores than the delayed group, regardless of sub-groups. Only in HI-averse group, the continued group compared to the delayed group significantly increased scores for premature response after acute cocaine injection. E and R represent extinction and re-acquisition, respectively. Values are expressed as a mean + standard error of mean. *p < 0.05, **p < 0.01; compared to the delayed group at each developmental period. ^*†*^*p* < 0.05, ^*††*^*p* < 0.01, ^*†††*^*p* < 0.001; compared to the basal score within either the continued or the delayed group.

Next, we examined how extinction, re-acquisition, and acute cocaine administration affect both premature responses and preference scores during rGT. During the extinction period, premature response scores rapidly decreased to nearly zero in both continued and delayed groups. Interestingly, however, during the re-acquisition period, rats in the continued group showed significant increase in premature responses compared with the delayed group, regardless of pre-selected types of impulsivity or preference (Figure 4). For the LI-averse group, results of the two-way repeated-measures ANOVA showed significant effects of age at exposure to rGT [continued vs. delayed; *F*_(1, 8)_ = 9.07, *p* = 0.017] and different experimental periods [basal vs. acute cocaine administration; *F*_(7, 8)_ = 6.76, *p* < 0.001]. *Post hoc* Bonferroni comparisons revealed that the rats in the continued group showed premature response significantly more often (*p* < 0.05–0.01) than those in delayed group on re-acquisition days 1, 4, and 7. For the HI-averse group, results of the two-way repeated-measures ANOVA showed significant effects of different experimental period [*F*_(7, 12)_ = 26.08, *p* < 0.001] and age at exposure to rGT × different experimental period interaction [*F*_(7, 84)_ = 3.01, *p* = 0.007]. *Post hoc* Bonferroni comparisons revealed that the rats in the continued group had significantly higher (*p* < 0.01–0.05) premature response scores than those in the delayed group; the continued group also had higher scores than their basal scores (*p* < 0.001–0.05), on re-acquisition days 1 and 7. For the LI-seeking group, results of the two-way repeated-measures ANOVA showed significant effects of different experimental period [*F*_(7, 13)_ = 12.80, *p* < 0.001]. *Post hoc* Bonferroni comparisons revealed that the rats in the continued group had significantly higher premature response scores (*p* < 0.05) than those in the delayed group on re-acquisition days 4 and 7. For the HI-seeking group, results of the two-way repeated-measures ANOVA showed significant effects of age at exposure to rGT [*F*_(1, 12)_ = 5.39, *p* = 0.039] and different experimental period [*F*_(7, 12)_ = 12.44, *p* < 0.001]. *Post hoc* Bonferroni comparisons revealed that the rats in the continued group showed premature response significantly more often (*p* < 0.01–0.05) than those in the delayed group on both re-acquisition days 4 and 7. Finally, after acute cocaine administration, rats in the continued group showed significant increase of premature responses than those in the delayed group; however, this effect appeared only in the HI-averse group (Figure 4). *Post hoc* Bonferroni comparisons revealed that the rats in the continued group showed significantly higher premature response scores than those in the delayed group (*p* < 0.05); the continued group also showed higher scores than their basal scores (*p* < 0.001) after acute cocaine administration.

**Figure 5:**
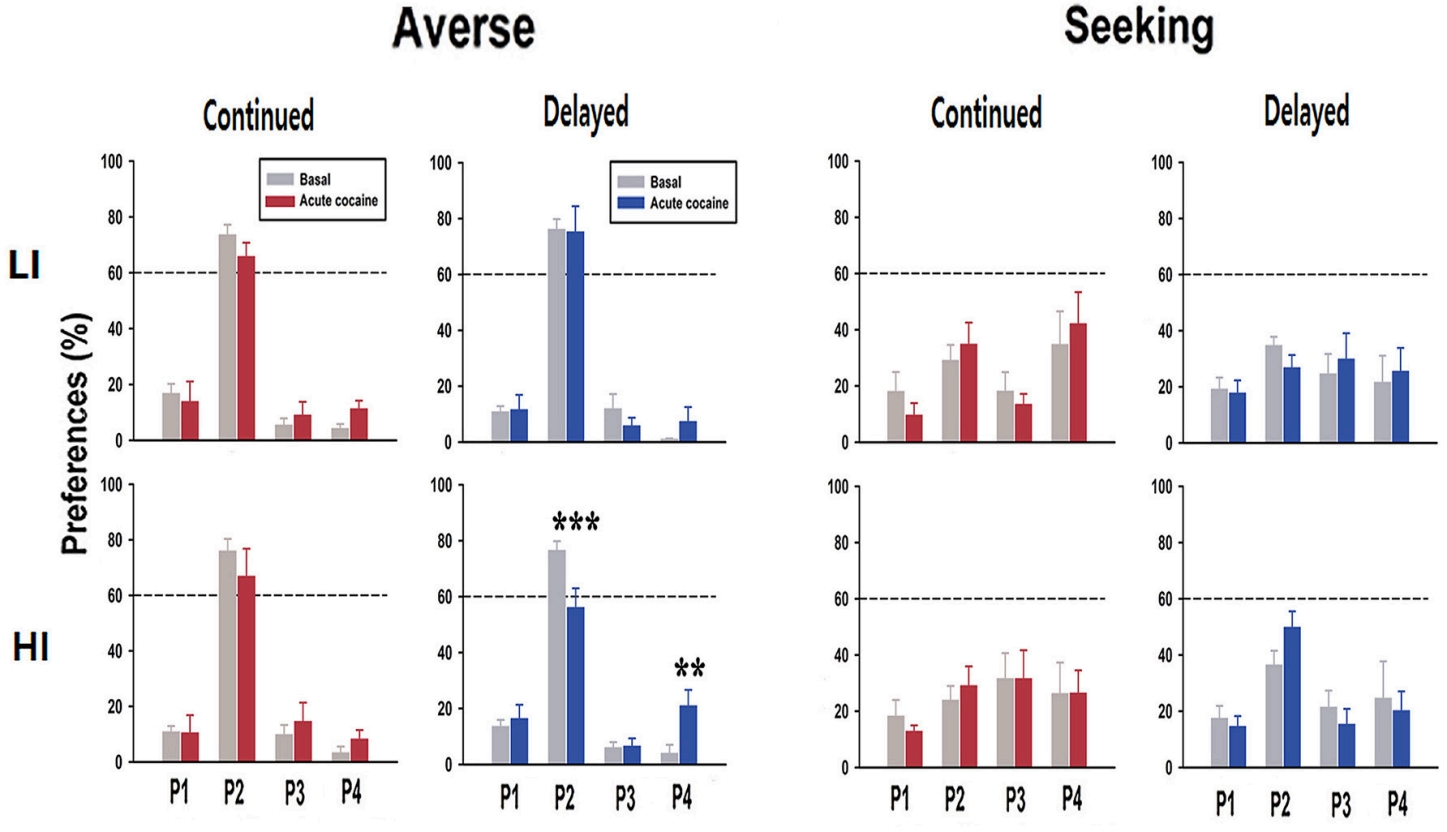
Preference scores after acute cocaine injection. In response to acute cocaine injection, rats only in the HI-averse-delayed group showed more risk-seeking choice preference (i.e., they chose P2 less and P4 more). Values are expressed as a mean + standard error of mean. ^**^p < 0.01, ^***^p < 0.001; compared to the basal score within either P2 or P4.

In contrast to the premature responses, the change of preference toward risk-seeking behavior was observed only when rats were acutely administered cocaine (Figure 5). Interestingly, among all the different combinations of groups, only in the HI-averse-delayed group, there were significant effects of drug [basal and acute cocaine; *F*_(1, 6)_ = 8.93, *p* = 0.024], window (P1, P2, P3, and P4; *F*_(3, 6)_ = 42.51, *p* < 0.001), and drug×window interaction [*F*_(3,18)_ = 10.93, *p* < 0.001]. *Post hoc* Bonferroni comparisons revealed that the rats acutely administered cocaine chose P2 significantly less often (*p* < 0.001), but chose P4 significantly more often (*p* = 0.002) than their basal preferences.

### Analysis of behavioral parameters

We further analyzed several choice-related behavioral parameters (10, 24) from the data obtained for the different experimental period. Overall, acute cocaine injection decreased choice response ratios compared with basal score in the following sub-groups; HI-averse-continued, HI-averse-delayed, LI-seeking-continued, LI-seeking-delayed, and HI-seeking-delayed (Tables 1A, B). Remarkably, both omission and reward collection latency were significantly increased, compared with the basal score, after acute cocaine administration only in the HI-averse-delayed sub-group (Table 1A). Two-way repeated-measures of ANOVA in these data showed significant effects of age at exposure to rGT [*F*_(1, 12)_ = 4.79, *p* = 0.049] and different experimental period [*F*_(3, 12)_ = 4.56, *p* = 0.008] for omission, and showed significant effect of different experimental period [*F*_(3, 12)_ = 3.31, *p* = 0.031] for reward latency. *Post hoc* Bonferroni comparisons of these data revealed that the rats in the delayed group exhibited significantly higher omission and reward latency (*p* < 0.01–0.05) than those in the continued group.

**Table 1A:** Analysis of behavioral parameters in the risk-averse groups.

**Group**		**Period**	**Choice response**	**Omission (%)**	**Perseverative response**	**Correct response latency (sec)**	**Reward collection latency (sec)**	**Feed-tray entry**
**LI**	**Continued**	Basal	60.40 ± 3.04	7.80 ± 1.64	1.40 ± 0.16	2.66 ± 0.16	2.59 ± 0.33	0.63 ± 0.13
		R1	53.80 ± 4.86	13.65 ± 4.23	1.51 ± 0.16	2.69 ± 0.41	3.32 ± 0.83	0.76 ± 0.07
		R7	59.80 ± 7.57	7.01 ± 1.73	1.65 ± 0.28	2.36 ± 0.19	2.88 ± 0.63	0.64 ± 0.07
		Acute coc	34.20 ± 9.26	20.38 ± 9.02	1.07 ± 0.22	2.78 ± 0.13	3.02 ± 0.51	0.35 ± 0.05
	**Delayed**	Basal	63.80 ± 8.33	7.39 ± 1.83	1.25 ± 0.18	2.81 ± 0.29	1.74 ± 0.17	1.19 ± 0.15
		R1	59.00 ± 3.70	13.18 ± 1.76	1.35 ± 0.23	3.45 ± 0.18	1.47 ± 0.07	0.99 ± 0.21
		R7	41.20 ± 5.03	11.19 ± 2.11	1.34 ± 0.35	2.98 ± 0.26	1.49 ± 0.08	1.26 ± 0.24
		Acute coc	40.20 ± 12.25	28.45 ± 10.56	1.50 ± 0.35	2.84 ± 0.57	3.49 ± 1.88	0.89 ± 0.18
**HI**	**Continued**	Basal	86.14 ± 3.16	2.39 ± 0.31	1.22 ± 0.13	1.81 ± 0.17	2.46 ± 0.81	1.07 ± 0.41
		R1	83.14 ± 6.95	2.61 ± 0.70	1.20 ± 0.22	1.86 ± 0.17	2.61 ± 1.11	1.66 ± 0.53
		R7	90.57 ± 5.43	0.78 ± 0.42	1.23 ± 0.17	1.58 ± 0.14	1.89 ± 0.18	1.49 ± 0.70
		Acute coc	55.29 ± 9.69^***^	7.42 ± 3.31	1.39 ± 0.19	2.01 ± 0.38	2.84 ± 0.51	0.73 ± 0.10
	**Delayed**	Basal	71.10 ± 6.10	3.64 ± 1.10	1.32 ± 0.22	2.15 ± 0.25	2.39 ± 0.53	0.77 ± 0.09
		R1	67.14 ± 10.07	8.81 ± 4.11	1.50 ± 0.35	2.57 ± 0.37	2.35 ± 0.54	1.21 ± 0.22
		R7	60.14 ± 5.73^††^	5.13 ± 1.50	0.93 ± 0.14	2.31 ± 0.26	2.88 ± 1.35	0.99 ± 0.23
		Acute coc	31.00 ± 4.15^***^^,^^†^	19.56 ± 7.74^**^^,^^†^	1.72 ± 0.35	2.63 ± 0.29	9.00 ± 3.26^**^^,^^††^	0.48 ± 0.10

*Each parameter's units of measurement are as follows: choice response (%), omission (%), perseverative response (actual number of touch), correct response latency (sec), reward collection latency (sec), feed tray entry (the number of feed-tray entries during ITI divided by the total number of trials initiated)*.

** *p < 0.01*,

*** *p < 0.001; compared to the basal score within either continued or delayed group*.

† *p < 0.05*,

†† *p < 0.01; compared to the continued group after acute cocaine injection*.

**Table 1B:** Analysis of behavioral parameters in the risk-seeking groups.

**Group**		**Period**	**Choice response**	**Omission (%)**	**Perseverative response**	**Correct response latency (sec)**	**Reward collection latency (sec)**	**Feed-tray entry**
**LI**	**Continued**	Basal	50.86 ± 5.21	5.36 ± 1.70	1.75 ± 0.17	2.06 ± 0.22	23.59 ± 8.14	0.89 ± 0.16
		R1	53.71 ± 4.06	9.09 ± 2.53	1.81 ± 0.41	2.45 ± 0.20	18.82 ± 8.92	1.03 ± 0.26
		R7	50.43 ± 4.70	6.37 ± 3.52	1.94 ± 0.27	1.98 ± 0.29	21.58 ± 9.31	0.94 ± 0.15
		Acute coc	34.71 ± 3.85^*^	19.08 ± 10.49	1.74 ± 0.28	2.07 ± 0.33	31.34 ± 11.39	0.65 ± 0.10
	**Delayed**	Basal	60.54 ± 6.04	4.49 ± 1.55	1.65 ± 0.16	1.96 ± 0.15	12.47 ± 4.56	1.25 ± 0.12
		R1	54.25 ± 6.89	13.91 ± 3.74	1.76 ± 0.33	2.68 ± 0.29	10.20 ± 3.51	1.58 ± 0.23
		R7	48.13 ± 6.98	6.26 ± 2.23	1.84 ± 0.29	2.58 ± 0.24	12.46 ± 4.36	1.37 ± 0.19
		Acute coc	33.75 ± 4.06^***^	13.53 ± 4.47	1.72 ± 0.45	2.49 ± 0.39	13.57 ± 4.21	0.83 ± 0.11
**HI**	**Continued**	Basal	54.05 ± 4.85	1.39 ± 0.47	2.21 ± 0.23	1.49 ± 0.15	20.96 ± 7.47	1.18 ± 0.41
		R1	54.43 ± 6.51	5.90 ± 2.05	1.77 ± 0.24	1.90 ± 0.24	20.80 ± 8.89	2.19 ± 0.69
		R7	59.14 ± 6.49	1.26 ± 0.81	2.32 ± 0.40	1.54 ± 0.21	20.73 ± 10.13	1.81 ± 0.84
		Acute coc	39.86 ± 4.78	5.42 ± 1.37	2.26 ± 0.25	1.56 ± 0.14	9.62 ± 2.18	0.63 ± 0.11
	**Delayed**	Basal	66.33 ± 7.62	2.34 ± 0.62	1.82 ± 0.24	1.80 ± 0.14	15.26 ± 8.26	0.66 ± 0.13
		R1	52.43 ± 8.45	11.29 ± 3.92	2.20 ± 0.59	2.52 ± 0.28	9.59 ± 5.87	0.73 ± 0.07
		R7	52.00 ± 7.92	2.76 ± 1.07	1.78 ± 0.27	2.09 ± 0.30	17.05 ± 8.66	0.76 ± 0.11
		Acute coc	44.00 ± 7.80^*^	16.55 ± 10.86	1.63 ± 0.43	2.36 ± 0.53	7.69 ± 2.85	0.62 ± 0.06

*Each parameter's units of measurement are as follows: choice response (%), omission (%), perseverative response (actual number of touch), correct response latency (sec), reward collection latency (sec), feed tray entry (the number of feed-tray entries during ITI divided by the total number of trials initiated)*.

* *p < 0.05*,

*** *p < 0.001; compared to the basal within either continued or delayed group*.

## Discussion

The present results clearly show that impulsive action was strongly increased in rats exposed early to rGT, as young adults (continued), compared with those exposed late to rGT, as mature adults (delayed), during re-acquisition after extinction in all sub-groups. Further, our results reveal that, although rats in the continued group have no difference in the preference scores in rGT after acute cocaine administration compared with their basal scores, those in the delayed group with HI-averse characteristics exhibit altered preferences, resulting in decreased preference for P2 and a simultaneously increased preference for P4. This is the first direct demonstration, to our knowledge, that two distinct subtypes of impulsivity can be differentially manifested depending on the developmental period at which the animals were first exposed to rGT.

In the present study, we adopted the methods previously introduced (23), with the slight modification of pre-selecting rats showing high and low impulsive action. Similar to the previous study, when we applied a rule of 7 sec for ITI, rats showed premature responses, with higher overall scores, and the differences between the HI and LI groups were amplified. These results indicate that there are inherent individual differences in rats for responding prematurely, consequently revealing their traits as HI or LI.

Although detailed studies on the relationship between impulsive action and impulsive choice are still scarce, it has previously been shown, using a within-subjects approach in rats, that they are not correlated; in this study, 5-CSRTT and a delayed reward task were used to measure impulsive action and impulsive choice, respectively (25). Consistent with these results, we found that rats pre-selected as HI and LI both showed similar level of preference toward dis-advantageous choice in rGT, regardless of the age at which they were exposed to rGT (Figure 3A). This is interesting because there is a similarity between the experimental scheme of the previous study and ours, in that both studies were conducted with a within-subjects approach using two different behavioral measurements continuously within the same subjects. In our study, stage 4 during pre-training, which is equivalent to 5-CSRTT in terms of basic concept and procedure, measures impulsive action, while stage 6, which is the main training for rGT, measures impulsive choice. These results indicate that difference at the level of impulsive action does not affect the appearance of impulsive choice later, at least in absence of external disturbances (e.g., stressful environment), supporting the notion that they are separable and distinct forms of impulsivity.

Nonetheless, it is often considered that one form of impulsivity (impulsive action) contributes to the development of disorders related to decision-making, mostly by enhancing the other form of impulsivity (impulsive choice) (26). Notably, it has been demonstrated that pre-selected HI rats are more prone to the development of compulsive drug taking even in the face of aversive outcomes (23, 27). Moreover, behavioral addiction, for example, gambling disorder, which is a typical manifestation of disorder with high impulsivity choice, is known to be associated with impulsive action (28). These results suggest that impulsive action somehow contributes to the appearance of impulsive choice, especially in an environmental setting with drugs of abuse (the former) and an unknown stressful situation (e.g., financial difficulty; the latter). Similarly, in the present study, when HI rats in the delayed group, previously categorized as risk-averse, were administered a single dose of cocaine after experiencing extinction and re-acquisition, they showed increased preference toward risk-seeking, which was not observed in LI rats (Figure 5). These results support the hypothesis that high impulsive action potentially contributes to the increased chance of making an impulsive choice when subjects are under the influence of drugs of abuse or/and stressful situation.

Further analysis of several choice-related behavioral parameters (10, 24) from the data obtained after acute cocaine injection revealed an interesting finding. Remarkably, only in the sub-group which showed preference change toward risk-seeking, i.e., HI-averse-delayed group, the reward collection latency was significantly higher, when either compared with the continued group after acute cocaine administration or the basal score of the same sub-group (Table 1). The higher reward collection latency may indicate that rats were more interested in an object, for example, the screen (or light on the screen) in this case, other than the pellet reward itself, consequently resulting in increased latency in collecting the reward. As shown in our previous study, there is a positive correlation between reward collection latency and disadvantageous choice in rGT (12); the higher reward collection latency observed in the HI-averse-delayed group may further indicate that this sub-group is more likely to be in the process of moving toward risk-seeking behavior, consistent with their actual decreased and increased preference scores for P2 and P4, respectively (Figure 5).

In contrast with the delayed group, the HI-averse-continued group did not exhibit significant change in the preference toward risk-seeking behavior even after acute cocaine administration (Figure 5). Instead, they showed conspicuous increase in premature responses compared with their basal scores, during re-acquisition as well as after acute cocaine administration (Figure 4). Although there were differences in the strength of the data, a similar trend, i.e., higher premature responses in continued than in delayed groups, also appeared throughout the other sub-groups (LI-averse, LI-seeking, and HI-seeking). These results show that rats in the continued group remained strongly consistent with their inherent trait rather than altering their behavior toward that of another subtype, i.e., impulsivity choice, as opposed to the delayed group.

Interestingly, a previous study showed that adolescent rats exposed to stress hormone exhibited reduced impulsive action but increased impulsive choice (15), which vaguely hints at the factors that differentially influence impulsive action and impulsive choice depending on the situation. However, our experimental schemes differ from those employed in the aforementioned study, with the rats in the continued group being exposed to rGT at ~10 weeks of age, which is equivalent to the late adolescent/young adult stage, while those in the delayed group were exposed to rGT at ~13 weeks, which is equivalent to the mature adult stage (13, 16). In order to see if there is any potential impact on the results by such interruption in performance, rats in delayed group performed stage 4 briefly again for 3 days just before entering into stage 5. Interestingly, it was verified that they all successfully passed the criterion for more than 80% of accuracy and <20% of omission scores, except two, which showed more than 20% in omission at this stage, but later back to <20% of omission score stably during stage 5 and thereafter. These results show that rats in delayed group still remember and are able to perform with no difficulty even with 3 weeks of interruption in performance. Thus, we can speculate that it is the difference in the age, a developmentally sensitive period, at first exposure to rGT that may somehow contribute to differential expression of the two subtypes of impulsivity. At present, we have no satisfactory explanation as to how all the differences in our results manifested.

In conclusion, our data clearly indicate that impulsive action and choice are distinct aspects of impulsivity, which are differentially influenced in rats by the age at the first exposed to gambling task. Our data also demonstrate that the differences may not be evident, and in order to resolve the two components, rats must be exposed to a stressful situation (e.g., extinction and subsequent re-acquisition) and/or drugs of abuse (e.g., acute cocaine injection). Finally, the mechanism through which the brain affects this process of differential influence of developmental periods on impulsivity remains largely unknown. More studies will certainly be conducted on these interesting phenomena in the future.

## Author contributions

BC, WK, and J-HK designed the experimental strategy and analyzed the data. BC, MK, and WK performed all the experiments. All author prepared the figures and tables in the manuscript. J-HK wrote the manuscript. All authors commented on and approved the final version of the manuscript.

### Conflict of interest statement

The authors declare that the research was conducted in the absence of any commercial or financial relationships that could be construed as a potential conflict of interest.
